# Fractal Modeling and Fractal Dimension Description of Urban Morphology

**DOI:** 10.3390/e22090961

**Published:** 2020-08-30

**Authors:** Yanguang Chen

**Affiliations:** Department of Geography, College of Urban and Environmental Sciences, Peking University, Beijing 100871, China; chenyg@pku.edu.cn

**Keywords:** fractal, fractal dimension, pre-fractal, multifractals, scaling range, entropy, spatial correlation, fractal cities

## Abstract

The conventional mathematical methods are based on characteristic length, while urban form has no characteristic length in many aspects. Urban area is a scale-dependence measure, which indicates the scale-free distribution of urban patterns. Thus, the urban description based on characteristic lengths should be replaced by urban characterization based on scaling. Fractal geometry is one powerful tool for the scaling analysis of cities. Fractal parameters can be defined by entropy and correlation functions. However, the question of how to understand city fractals is still pending. By means of logic deduction and ideas from fractal theory, this paper is devoted to discussing fractals and fractal dimensions of urban landscape. The main points of this work are as follows. Firstly, urban form can be treated as pre-fractals rather than real fractals, and fractal properties of cities are only valid within certain scaling ranges. Secondly, the topological dimension of city fractals based on the urban area is 0; thus, the minimum fractal dimension value of fractal cities is equal to or greater than 0. Thirdly, the fractal dimension of urban form is used to substitute the urban area, and it is better to define city fractals in a two-dimensional embedding space; thus, the maximum fractal dimension value of urban form is 2. A conclusion can be reached that urban form can be explored as fractals within certain ranges of scales and fractal geometry can be applied to the spatial analysis of the scale-free aspects of urban morphology.

## 1. Introduction

Scientific research starts from description of a phenomenon and then focuses on understanding its work principle. The simple description is based on measurements, while the complex description relies heavily on mathematical methods [1]. In order to describe a city, we try to express it using data. Mathematical description depends on measurement description, as measurement can be treated as the basic link between mathematics and empirical studies [2]. In order to show the results from a measurement, we should find the characteristic scale of an entity. A characteristic scale is a special one-dimensional measure and can be termed characteristic length, which can integrate a great number of values into a simple number. Unfortunately, in many cases, it is impossible to find a characteristic length to describe a complex system such as a city and a system of cities. If so, we should substitute scaling concept for the characteristic scale concept. Fractal geometry can be regarded as one of the best mathematical tools for scaling analysis at present.

What is a fractal? This is not a problem for many scientists who are familiar with fractals. A fractal is regarded as a shape that is made of parts similar to the whole in some way [3]. Quantitatively, a fractal is defined as a set for which the Hausdorff–Besicovitch dimension is strictly greater than the topological dimension [4]. These definitions are suitable for the classical fractals, which belong to a group called thin fractals. The general concept of fractals is well known, but the question of how to understand fractals is still a problem for specific subjects such as urban geography. A fractal has no characteristic scale and cannot be described with traditional measures such as length, area, volume, and density. The basic parameter used for fractal description is fractal dimension. Since the length of coastline cannot be effectively measured, Mandelbrot put forward the concept of fractal dimension [5]. Fractal dimension can be defined on the basis of entropy and correlation function [3,4,6]. It is actually the invariant quantity in scaling transform and thus a parameter indicating symmetry. Where there is an immeasurable quantity, there is symmetry [7]. The discovery of fractals is essentially a discovery of scaling symmetry, namely the invariance under contraction or dilation transformation [8]. The immeasurability of the length of coastline enlightened Mandelbrot to think about the problem of contraction–dilation symmetry [5].

Cities and networks of cities are complex systems bearing the property of scaling symmetry. In urban studies, it is impossible to determine the length of the urban boundary and the area within the urban boundary objectively [9,10]. In this case, it is impossible to quantify the population size of a city. The precondition of determining urban population size is to determine the urban boundary line effectively. Population is one of the central variables in the study of spatial dynamics of city development [11], and it represents the first dynamics of urban evolution [12]. If we cannot measure urban population size, how can we describe a city and measure levels of urbanization? If we cannot describe a city and quantify urbanization levels, how can we understand the mechanisms of urban evolution? Fortunately, today, we can employ the fractal dimension of urban form to replace urban area and urban population size. However, a new problem has emerged: how can we define a city fractal and determine its fractal dimension? Although fractal cities have been studied for more than 30 years, some basic problems still puzzle many theoretical geographers. This paper is devoted to answering these questions in terms of the author’s experience of long-term studies on fractal cities.

## 2. Fractal Cities and City Fractals

### 2.1. Are Cities Fractals

Is the coast of Britain a real fractal line? In fact, we cannot find any real fractals (based on fractal geometry) in the real world. This is similar to the fact that we cannot find circles and triangles (based on Euclidean geometry) in the real world. All of the fractal images that we encounter in books and articles represent pre-fractals rather than real fractals in the mathematical sense. A real fractal has infinite levels, which can only be revealed in the mathematical world, but a pre-fractal is a limited hierarchy indicating a fractal-like geometric form, which can be found in any textbooks on fractals. We can use the ideas from fractal geometry to research pre-fractals, including regular pre-fractals and random pre-fractals. The coast of Britain can be regarded as a random pre-fractal curve instead of a real fractal line. However, we can study the coast of Britain using the ideas from fractals and fractal dimension. Similarly, cities are not true fractals but proved to be random pre-fractals because urban form has no characteristic scales. A great number of empirical studies show that, based on certain scaling ranges, urban form satisfies three necessary and sufficient conditions for fractals (Table 1). Urban form follows power laws, which indicates that cities can be treated as pre-fractals. The basic property of a random pre-fractal object is that its scaling range is limited, and its fractal dimension value is based on the scaling range [13].

### 2.2. Fractal Geometry: An Approach to Scale-Free Analysis

Fractal geometry is a powerful tool for scaling analysis of scale-free phenomena such as urban form. Scaling suggests that there is no characteristic scale in an entity. Cities, in many aspects, have no characteristic scale and cannot be effectively modeled by conventional mathematical methods. In contrast, urban phenomena can be well characterized by fractal parameters. Natural and social phenomena can be roughly divided into two categories: one is the phenomena with characteristic scales, and the other is the phenomena without characteristic scales. The former can be termed scaleful phenomena, and the later can be termed scale-free phenomena (Table 2). For the scaleful phenomena, we can find definite length, area, volume, density, eigenvalue, mean value, standard deviation, and so on. If the spatial distribution of this kind of phenomena is converted into a probability distribution, it has clear and stable probability structure and thus can be described with Gaussian function, exponential function, logarithmic function, lognormal function, Weibull function, etc. The conventional higher mathematics can be used as an effective tool for modeling and analyzing such phenomena. On the contrary, for the scale-free phenomena, we cannot find effective length, area, volume, density, eigenvalue, mean value, standard deviation, and so forth. If the spatial distribution of this sort of phenomenon is transformed into a probability distribution, it can be characterized with power functions, Cobb–Douglas function (production function), or some types of function including hidden scaling. The probability structure of the scale-free distributions is not certain. Traditional advanced mathematics cannot effectively characterize such phenomena. In recent years, a number of theoretical tools for scale-free analysis have emerged, including fractal geometry, wavelet analysis, allometric theory, and complex network theory. Among various “new” tools, fractal geometry represents an excellent method for scale-free modeling and scaling analysis.

A city is a complex system with multifaceted characteristics. In some respects, a city has characteristic scales, e.g., urban population density distribution, which follows negative exponential law and can be described with Clark’s model [14]. The spatial distribution function can be derived from the principle of entropy maximization [15]. In another respect, a city has no characteristic scale, e.g., urban traffic network density distribution, which follows inverse power law and can be characterized with Smeed’s model [16]. The corresponding spatial distribution can be characterized by spatial correlation and allometric scaling [17]. Where land use is concerned, urban form follows the power law distribution and can be treated as random pre-fractal patterns [9,10]. In this sense, we cannot find effective characteristic scales for urban morphology. Consequently, the traditional methods of quantitative analysis and mathematical modeling are often invalid for research on urban form and growth. As a substitute, fractal geometry is one of feasible mathematical tools for the spatial analysis of cities.

### 2.3. How to Define City Fractals

The angle of view for fractal studies of cities depends on the definition of embedding space. A city fractal based on digital maps or remote sensing images can be defined in a two-dimensional embedding space, and also it can be defined in a three-dimensional embedding space [18]. Generally speaking, fractal cities are defined in a two-dimensional embedding space based on digital maps or remote sensing images [9,10,19]. However, some scholars study fractal cities through three-dimensional embedding spaces [20]. The fractal city defined in a three-dimensional embedding space has attracted the attention of geographers [18]. In fact, a fractal based on the three-dimensional embedding space can be explored through the two-dimensional embedding space. In the simplest case, the relationship between the fractal dimension based on two-dimensional embedding space, *D*^(2)^, and the fractal dimension based on three-dimensional embedding space, *D*^(3)^, is as follows, *D*^(3)^ = 1 + *D*^(2)^ [21].

For simplicity, we define the city fractals in a two-dimensional embedding space. The main reasons are as follows.

Firstly, fractal dimension is used to replace the two-dimensional urban area rather than the three-dimensional urban volume. In order to study a city, we must describe a city; in order to describe a city, we must know its basic measures such as population size, urban area, and economic output. Unfortunately, urban form has no characteristic scales due to its fractal properties, and thus the urban boundary cannot be objectively determined. Urban area cannot be objectively calculated because the measurement results depend on scales. This is the well-known scale-dependence property of urban form; the cause lies in scale-free distribution of urban land use. In this case, the fractal dimension of urban form can be employed to replace the urban area to reflect the extent of space filling. The fractal dimension as a degree of urban space filling is exactly a substitute of the urban area. Urban area is a scale-dependent measure, while fractal dimension is scaleful parameter. In this sense, fractal dimension is more effective than urban area to reflect urban spatial development. Incidentally, some scholars prefer to define a city fractal in a three-dimensional space—this means that they try to calculate a fractal dimension based on three-dimensional embedding space to replace urban volume.

Secondly, the general principle of model building is based on reduction of dimension. The effective skill of scientific quantitative analysis is to reduce dimension instead of increasing dimension. The basic relation between spatial dimension *n* and the degree of analytical complexity *C* can be expressed as *C* = *n*(*n* − 1)/2, which represents the least statistical parameter number for quantitative analysis. The well-known Clark’s law of urban population density distribution in a two-dimensional space is actually based on a one-dimensional space modeling, but this model reflects the geographical information in a three-dimensional space [14]. In other words, the population distribution in the three-dimensional space is projected onto the two-dimensional space by population density, and then the mathematical expression is established on the basis of the one-dimensional space with the help of statistical averaging [15]. The same is the case with Smeed’s model of urban traffic density distribution [9,16,17]. If we study a city fractal through a three-dimensional embedding space, the amount of work and difficulty of fractal dimension calculation is considerably increased, and the accuracy of fractal parameter estimation is reduced, but the increment of the gained geographic information is very limited. In short, it is hard to promote the analytical effect of fractal cities significantly by substituting the two-dimensional embedding space with the three-dimensional embedding space.

Thirdly, the allometric scaling relation between population and land use suggests that urban form should be defined in a two-dimensional space. The allometric scaling exponent *b* is the ratio of the fractal dimension of urban form *D*_f_ to the dimension of urban population *D*_p_; that is, *b* = *D*_f_/*D*_p_. Empirical studies show that the *b* values are close to 0.85 [22]. If *D*_f_ > 2, then we have *D*_p_ > 2/0.85 = 2.35. Based on Clark’s law and scaling analysis, urban population distribution proved to be a two-dimension phenomenon (*D*_p_ = 2) [23]. If the urban form is defined in a three-dimensional embedding space, the fractal dimension *D*_f_ values will be between 2 and 3, and the allometric scaling exponent *b* values will be greater than 1. However, the observational values of allometric scaling exponent *b* range from 2/3 to 1 in most cases; that is, 2/3 < *b* < 1 [22,24,25]. This suggests that the dimension of urban form, *D*_f_, is between 1 and 2. In fact, in urban studies, fractal dimension is not a concept of comparability. The fractal dimension value depends on the definition of embedding space.

If a city fractal is defined in a two-dimensional embedding space, the fractal form includes two aspects: urban area and urban boundary. The above discussion is actually based on urban area, but urban boundary can be treated as fractal lines [9,26,27,28,29,30,31]. The closed urban boundary curve is termed the urban envelope, in which we can determine a Euclidean urban area [9,32]. The length of the urban boundary and the Euclidean area within the urban envelope follow the geometric measure relation as follows:

(1) $$A=aL^{2/D_{b}}$$

where *A* refers to the Euclidean area of a city (urban area), *L* denotes the length of urban envelope (urban perimeter), *a* is the proportionality coefficient, and *D*_b_ is the fractal dimension of urban boundary, which can be termed boundary dimension [28]. In fact, Equation (1) can be generalized to the more general expression shown below [27,33]:

(2) $$A=aL^{D_{f}/D_{b}}$$

where *A* denotes the Euclidean area of a city, and *D*_f_ is the fractal dimension of “urban area”. Equation (2) is in fact an allometric scaling relation of urban shape [33]. The topological dimension of the urban boundary is *d*_T_ = 1, so the boundary dimension is greater than 1. The fractal parameter value is between 1 and 2; that is, 1 < *D*_b_ < 2. Now, a question arises: what determines the lower limit of fractal dimension of urban morphology, urban area or urban boundary? The answer is clear. If we study urban form and try to substitute the urban area with form dimension, it is the topological dimension of the urban area that determines the least value of the fractal dimension; on the other hand, if we research urban boundary and attempt to replace urban perimeter length with boundary dimension, it is the topological dimension of urban boundary that determines the minimum value of the fractal dimension. In most cases, we study an urban impervious area which is represented by the pixels of buildings (fractal separated spaces) rather than the urban boundary (fractal lines).

### 2.4. The Lower and Upper Limits of Fractal Dimension

Fractal dimension values have a strict lower limit and upper limit. This is beyond doubt. However, what are the lower limit and upper limit of the fractal dimension of urban from? This is still a pending question. Empirically, if a city fractal is defined in a two-dimensional embedding space, the fractal dimension value is between 0 and 2 [34,35,36,37]. In theory, the lower and upper limits of the fractal dimension of urban form rely on the topological dimension and embedding dimension. In many cases, the box-counting method is employed to estimate the fractal dimension values of urban form. The lower limit of the fractal dimension *D*_min_ depends on the topological dimension of urban form *d*_T_, while the upper limit *D*_max_ depends on the Euclidean dimension of the embedding space *d*_E_. As indicated above, the embedding space can be defined as a two-dimensional space; thus, the Euclidean dimension of *d*_E_ = 2, so we have *D*_max_ ≤ *d*_E_ = 2. As for the topological dimension of urban form, *d*_T_, in theory, it should be *d*_T_ = 0. Therefore, we have *D*_min_ ≥ *d*_T_ = 0.

How can we determine the topological dimension of urban form? As we know, the Lebesgue measures of real fractals are zero [4]. This suggests that if we treat urban form as a fractal, the urban area of land use should be treated as zero. Please note that this is based on theoretical understanding, which is different from reality. How can we understand the assumption that the area of a city fractal is zero? This means that an urban fractal can be reduced to either a separated space or a space-filling curve under the limit conditions. For a separated space, the topological dimension is *d*_T_ = 0, while for a space-filling curve, the topological dimension is *d*_T_ = 1. In fact, using the ArcGIS technique, we can reduce a city fractal to a separated space rather than a space-filling curve. A separated space of a city comprises pixels or building cells on a remote sensing image or digital map. This indicates that the topological dimension of city fractals is *d*_T_ = 0 instead of *d*_T_ = 1. According to Shen [36], the box dimension values of Baltimore are between 0.6641 and 1.7211 from years 1792 to 1992.

**Figure 1:**
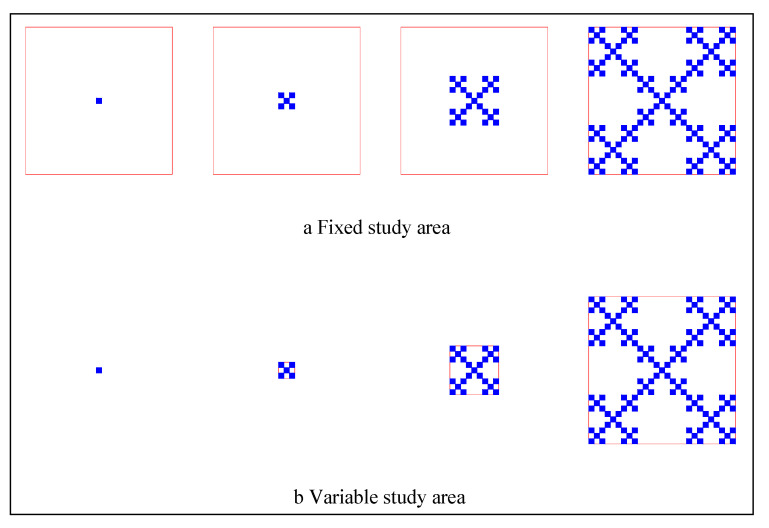
The sketch maps for two types of approaches to defining study areas for fractal dimension estimation of urban form (by Chen [34]). Note: The square frames surrounding the growing fractals represent the study area of fractal dimension measurements. Figure 1a shows a fixed study area, and Figure 1b displays a variable study area, the size of which depends on the extent of fractal city cluster.

In practice, the lower and upper limits of fractal dimension of urban form depend on the methods of defining the study area. There are two approaches to obtaining the time series of the fractal dimension values of urban growth and form [34]. One is based on a constant study area [9,36], and the other is based on a variable study area [19,37]. Each approach has its advantages and disadvantages (Table 3). If we define a study area with fixed size for different years, the largest box can be determined by the urban boundary of the most recent year. Then, the largest box can be applied to digital maps of the city in previous years (Figure 1a). Using the same set of boxes, we estimate the fractal dimension values of urban form in different years. Based on this approach, the fractal dimension values of a city’s form in different years are more comparable. The time series of fractal dimension values can better reflect the space replacement process of an urban region. The subsets of the time series are termed sample paths. If a sample path is very long, the original urban form can be treated as a point. As a result, the fractal values may be between 0 and 2 [34,38]. In contrast, if we define a variable study area, the size of the largest box is determined by the urban boundary in a given year. Thus, the largest boxes are different from year to year (Figure 1b). Based on this approach, the comparability of fractal dimension values of urban form in different years is reduced. However, these fractal dimension values can better reflect the degree of urban space filling. As a result, the fractal values may be between 1 and 2 [34].

## 3. Fractal Modeling of Urban Form

### 3.1. Two Research Directions of Fractal Cities

A complete scientific research process comprises two elements. One is to describe a system, and the other is to understand the mechanism by which the system works. In short, scientific studies should proceed first by describing how things work and later by understanding why [39]. Accordingly, the scientific method contains two elements: description and understanding. Concretely speaking, as stated by Henry [1] (p. 14), “The two main elements of this scientific method are the use of mathematics and measurement to give precise determinations of how the world and its parts work, and the use of observation, experience, and where necessary, artificially constructed experiments, to gain understanding of nature.” A comparison between the two elements of the scientific process can be drawn as follows (Table 4). The most important method of scientific description is to establish mathematical models.

Fractal theory comprises two related parts: one is the scaling theory of complex systems, and the other is the mathematical method known as fractal geometry. As a complex system theory, it can be employed to understand the complexity of cities; as a geometry, it can be used to describe cities from the angle of view of scaling analysis. In fact, a mathematical theory plays two roles in any type of scientific research (Table 5). One is to produce models and develop a theory (mathematical modeling), and the other is to process experimental and observational data (statistical analysis). In urban studies, fractal geometry can serve two functions. One is to establish models for cities as systems and systems of cities, and the other is to carry out empirical analysis of cities using observational data. Many scholars utilize fractal geometry to process the observational data of urban geography, but I emphasize the basic function: mathematical modeling. No matter what type of study is conducted, there is no contradiction between models and observed data. All models rely heavily on observational data. The spatial data can be used in the empirical analyses of fractal models of cities.

In fact, one of the main tasks in scientific research is to produce models. As Neumann [40] (p. 492) said, “The sciences do not try to explain, they hardly even try to interpret, they mainly make models.” I agree with Hamming [41], who said, “The purpose of modelling is insight, not numbers.” Karlin [42] has a similar viewpoint, “The purpose of models is not to fit the data, but to sharpen the questions.” However, the confidence level of a model depends heavily on the relationship between mathematical expression and observed data. In order to verify a mathematical model, we must fit it to observational data and illustrate the statistical relationships and analytical effect. I am very much in favor of the viewpoint of Louf and Barthelemy [43], who said, “The success of natural sciences lies in their great emphasis on the role of quantifiable data and their interplay with models. Data and models are both necessary for the progress of our understanding: data generate stylized facts and put constraints on models. Models on the other hand are essential to comprehend the processes at play and how the system works. If either is missing, our understanding and explanation of a phenomenon are questionable. This issue is very general, and affects all scientific domains, including the study of cities.” The basic functions of mathematical models are explanation and prediction. As Fotheringham and O’Kelly [44] pointed out, “All mathematical modelling can have two major, sometimes contradictory, aims: explanation and prediction.” Not only that, as Kac [45] observed, “The main role of models is not so much to explain or predict—although ultimately these are the main functions of science—as to polarize thinking and to pose sharp questions.” The chief uses of fractal models lie in explanation and prediction. Let us take the logistic model of fractal dimension growth curves as an example. The model can be used to explain the speed change characteristics of urban growth [35]. It can tell us when the growth rate of a city will peak. It can also tell us the maximum space-filling index of a city’s land use. What is more, the model can sharpen questions for us. For example, the similarity and difference between the model of fractal dimension growth curves of Chinese cities such as Beijing and that of cities in western countries such as London, Baltimore, and Tel Aviv gives rise to new thinking about the spatial dynamics of urban evolution.

### 3.2. Two Approaches to Modeling Cities

As indicated above, one of the important tasks of fractal urban studies is to produce models. As Longley [46] (p. 605) pointed out, “In the most general terms, a ‘model’ can be defined as a ‘simplification of reality’, nothing more, nothing less.” In scientific research, mathematical models can be classified into two categories: mechanistic models and parametric models [47]. Accordingly, there exist two approaches to establishing mathematical models: analytical methods and experimental methods [48] (Table 6). The so-called analytical method is the approach to deriving a mathematical model with the help of existing scientific theories and laws and in light of the relationship and evolution of the various components of the studied system. The process is as follows: establish a functional equation based on one or more postulates, and then find the general solution to the functional equation. The solution to the equation is exactly the theoretical model (mechanistic or structural model) that we need. The experimental method is to select the most appropriate model in a set of hypothetical or imaginary models so that the model can be well fitted to the observational or experimental data. What is more, the model will not give rise to logical contradiction and difficulty in interpretation. Thus, we have an empirical model (parametric or functional model). In geography, the traditional gravity model is an empirical model, which is obtained by analogy with Newton’s law of universal gravitation. In contrast, the spatial interaction model of Wilson [49] is a theoretical model. The model is derived by constructing the postulates and solving the maximum entropy equation of traffic flows. The two types of models are not opposed but can be transformed into each other. An effective theoretical model must be an empirical model, which must be well fitted to observational data. On the other hand, an empirical model will become a theoretical model by mathematical demonstration. A typical example is Clark’s urban population density model [14]. The model was originally presented as an empirical model based on observation data [9]. However, it has become a theoretical model because it can be derived from the postulates of spatial entropy maximization of urban population distribution [15]. In an article, limited to the conditions at the time, we may fulfil some aspects of the research work but not necessarily complete all the research processes.

### 3.3. Fractal Models and Parameters of Cities

We have at least three approaches to developing mathematical models of urban form by using ideas from fractal theory. The first is to produce new models, the second is to improve the old models, and the third is to borrow models from other disciplines (Table 7). A typical example is the models of the fractal dimension growth curve of urban form: different approaches result in different models, and different models are suitable for different situations [34,35]. It is necessary to briefly comment on the third approach. In scientific research, a mathematical model can be transplanted from one field and applied to another field. The logistic function was originally proposed by Verhulst in 1838 to predict population growth [50]. Today, the well-known logistic function has been employed to predict many growing phenomena in many different fields, including urbanization levels and fractal dimension growth [35]. Similarly, the Boltzmann equation can also be generalized to other fields and used to model urban growth [34,51]. The allometric growth equation of urban geography came from biology [28,52]. The gravity model of geography resulted from Newton’s law of universal gravitation by analogy, and the spatial autocorrelation models of geography come from mathematical and statistical biology. These examples are too numerous to enumerate. The uniqueness of different fields is always determined by the physical meaning of model parameters rather than by the expression of mathematical models. The mathematical expression of a model is often general, but the parameters are for special purposes. The same mathematical model can be applied to many different fields, but different fields have different parameter meanings.

The notion of maximum and minimum of fractal dimension discussed above is important for producing models of the fractal dimension growth curves of urban form. The fractal dimension growth curve results from the time series of urban growth. In theory, we can calculate the fractal dimension values of a city’s form at different times. These values compose a sample path of fractal dimension and further form a curve of fractal dimension change of urban morphology. A sample path can be regarded as a subset of a time series [53]. Due to the lower and upper limits of urban fractal dimension, a fractal dimension growth curve takes on a squashing effect and can be described with one of the sigmoid functions such as logistic function and Boltzmann’s equation [34,35,54]. On the other hand, the question of how to determine fractal parameter values depends on specific research objectives and data conditions. This is a complex problem and needs to be judged on the basis of long-term research experience. Even for theoretical research, if the sample path of fractal dimension is short, we can take *D*_min_ = 1 and adopt the quadratic Boltzmann equation. For example, in one of the studies conducted by Chen [35], the time span was around 25 years (1984–2008). All the fractal dimension values are greater than 1. On the other hand, even for application research, if the sample path of fractal dimension is very long, we can take *D*_min_ = 0 and adopt the quadratic logistic function. For instance, in the study of Shen [36], the time span was around 200 years (1792–1992). One of the fractal dimension values for early years was less than 1. The situations can be classified into four groups and tabulated as below (Table 8).

## 4. Questions and Discussion

### 4.1. Problems of Fractal Dimension Values

The concept of fractal dimension proceeded from Hausdorff’s fractional dimension. Today, there are various definitions for fractal dimension, and the common fractal dimensions in urban studies are the box dimension and similarity dimension. The box dimension is mainly suitable for the spatial structure of cities and systems of cities, while the similarity dimension is chiefly applied to urban hierarchies, including hierarchies of cities and hierarchies of urban internal elements such as land use patches. Generally speaking, fractal dimension values come between the topological dimension and the Euclidean dimension of embedding space. For a regular fractal, if fractal copies/units have no overlapping, the Hausdorff dimension will equal the similarity dimension. Empirically, both the Hausdorff dimension and similarity dimension can be represented with the box dimension. All these dimension values are less than the Euclidean dimension of the embedding space and greater than the topological dimension of fractal objects. However, if fractal copies have overlapped parts, the similarity dimension will exceed the dimension of embedding space in value. Thus, similarity dimension will not equal the Hausdorff dimension or box dimension. In contrast, the box dimension will never exceed the embedding dimension.

**Figure 2:**
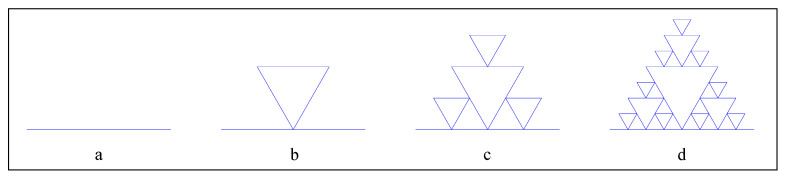
The interior boundary line of the Sierpinski gasket (the first four steps) (**a**) Initiator; (**b**) Generator; (**c**) The third step; (**d**) The fourth step.

**Figure 3:**
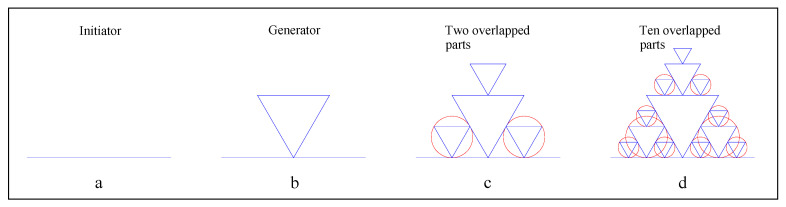
A special fractal line with overlapped parts (the first four steps). (**a**) Initiator; (**b**) Generator; (**c**) The third step; (**d**) The fourth step.

Let us examine two kinds of fractal dimension of the fractals with overlapped parts. The interior boundary line of the Sierpinski gasket is a typical fractal line with overlapped parts (Figure 2). The initiator is a straight-line segment with length of unit (Figure 3a); the generator is a curve consisting of five straight-line segments with the length of 1/2 unit (Figure 3b). From step 3 onward, fractal copies begin to overlap with one another, and the overlapped parts are marked with red circles (Figure 3c,d).

The similarity dimension and box dimension can be calculated by the ideas from fractal dimension. In the *m*th step, the length (linear size) of line segments can be expressed as

(3) $$s_{m}={\left(\frac{1}{2}\right)}^{m-1}$$

where *m* = 1, 2, 3, … denotes the ordinal numeration of steps. The number of line segments in each step can be counted in two different ways. One is to repeat the counting of the overlapped parts, and the other is to count the overlapped parts only once. For example, for the curve of step 3 (Figure 2c and Figure 3c), the number of line segments is *N*_3_ = 5^2^ = 25 according to the first counting method and *N*_3_ = 3 × 5 + 2^2^ = 19 according to the second counting method. According to the first method with repeated counting, the line segment number in the *m*th step is

(4) $$N_{m}=5^{m-1}$$

Thus, the similarity dimension is

(5) $$D_{s}=-\frac{\ln(N_{m+1}/N_{m})}{\ln(s_{m+1}/s_{m})}=\frac{\ln5}{\ln2}=2.322>d=2$$

According to the second method without repeated counting, the line segment number of step *m* is

(6) $$N_{m}=3N_{m-1}+2^{m-1}$$

where *N*_0_ = 0 for *m* = 1. By recurrence, we have

(7) $$N_{m}=\sum_{j=0}^{m-1}\left(3^{m-1-j}2^{j}\right)=3^{m-1}\sum_{j=0}^{m-1}\left[{\left(\frac{2}{3}\right)}^{j}\right]$$

where *j* = 1, 2, … *m*−1. Under the condition of limit, the result is

(8) $$N_{m}=\underset{m\to \infty }{\lim}\left[3^{m-1}\sum_{j=0}^{m-1}\left[{\left(\frac{2}{3}\right)}^{j}\right]\right]=3^{m-1}\frac{1}{1-2/3}=3^{m}$$

This suggests that when *m* becomes large enough, *N_m_* will approach 3*^m^*. Therefore, the box dimension is

(9) $$D_{b}=-\frac{\ln N_{m}}{\ln s_{m}}=\frac{m\ln3}{(m-1)\ln2}\overset{m\to \infty }{\to }\frac{\ln3}{\ln2}\approx 1.585<d=2$$

For this special regular fractal, the box dimension equals the Hausdorff dimension in theory. Therefore, for the regular monofractals with overlapped units, we have the following relation: topological dimension < Hausdorff dimension = box dimension < embedding space dimension < similarity dimension. However, for the regular monofractals without overlapped units, the dimension relation is as follows: topological dimension < Hausdorff dimension = box dimension = similarity dimension < embedding space dimension.

The phenomenon of overlapped fractal units resulting in fractal dimension values greater than the embedding space dimension can be employed to explain abnormal multifractal spectral curves. In theory, the generalized correlation dimension, *D_q_*, should be between 0 and 2 if a fractal city is defined in a two-dimensional embedding space. However, in many cases, the generalized correlation dimension values of urban morphology always exceed 2 or even go beyond 3 if the moment order, *q*, approaches negative infinity [55]. The reason is that, even based on the box-counting method, if *q* < 0 or *q* > 1, we will obtain the similarity dimension instead of a strict box dimension of urban form. When *q* < 0, the small patches in the urban pattern are enlarged gradually, and this leads to the overlapping and interlacing of random fractal units. If and only if the urban spatial structure is very well organized, the overlapping distributions of the magnified patches will be reduced in order to be omitted. In this sense, multifractal spectra can be adopted to appraise the quality of the spatial structures of cities and systems of cities.

### 4.2. Spatial Meanings of Fractal Dimension

Fractal dimension is a measure for scale-free phenomena which have no characteristic scales and cannot be effectively described by traditional mathematical methods. Where cities are concerned, the meanings and uses of fractal dimension of urban form rest with at least three aspects: degree of space filling, degree of spatial uniformity, degree of spatial complexity. As a space-filling index, fractal dimension can be used to reflect the replacement process of urban and rural space in theory. Unfortunately, it is both impossible and unnecessary to distinguish between urban area and rural area strictly. When we define a study area for a fractal cities, it comprises urban buildings, rural buildings, and other types of land. Various types of land form a hierarchy, with a cascade structure of land use based on different levels of scales [56]. In the urban regions, there are rural buildings, and in the rural regions, there are urban buildings. If we examine a city’s form from various spatial scales, we can find interlaced distributions of urban and rural land and buildings. The hierarchy with the cascade structure of urban and rural landscapes should be described with multifractals [57]. To solve this problem, we can use the concepts of space-filling extent, *U*(*t*), and space-saving extent, *V*(*t*), to replace urban land use and rural land use [34].

In the generalized correlation dimension spectrum, three parameters are very important, namely capacity dimension, information dimension, and correlation dimension. Among the three common parameters, capacity dimension is the most basic. The essence of capacity dimension is just space-filling ratio, and this can be demonstrated easily. Space-filling measures should be defined by logarithmic scales rather than conventional scales. The reason is that the spatial recursion process is based on exponential decay and logarithmic scale [9,57,58]. Let us define an index of space filling as follows:

(10) $$F=\frac{\ln A_{b}(r)}{\ln A(r)}=\frac{\ln N_{b}(r)}{\ln N(r)}$$

where *F* denotes the space-filling ratio, *A*_b_ refers to filled area, which can be represented by impervious area, *A* is the total area, *N*_b_ is the number of nonempty boxes, and *A* is the number of all boxes. It can be proven that

(11) $$2F=\frac{2\ln A_{b}(r)}{\ln A(r)}=\frac{2\ln N_{b}(r)}{\ln N(r)}=D_{0}$$

where *r* denotes the ordinal numeration of steps, and *D*_0_ refers to capacity dimension. For example, for the growing fractal displayed in Figure 1, we have

(12) $$2F=\frac{2\ln(5^{m})}{\ln(9^{m})}=\frac{2\ln(5)}{\ln(9)}=\frac{\ln(5)}{\ln(3)}=D_{0}$$

where *m* refers to the ordinal numeration of steps. This suggests that the doubling space-filling ratio yields the capacity dimension of a regular fractal. This conclusion can be generalized to urban morphology. On the other hand, in Equation (10), the numerator is the Hartley entropy, *H*, and the denominator is the maximum entropy, *H*_max_ [55]. If the minimum entropy and minimum fractal dimension are zero, the space-filling ratio is proven to be the normalized entropy and the normalized capacity dimension [59]. What is more, fractal dimension is proven to be the scaling exponent of spatial correlation, and a correlation function can be expressed as

(13) $$C(r)=C_{1}r^{2(D_{0}-d)+1}$$

where *r* refers to distance, *C*(*r*) denotes spatial correlation function, *C*_1_ is proportionality coefficient, and *d* represents embedding space dimension [60]. Spatial correlation suggests spatial displacement, which corresponds to time lag and implies spatial complexity. In short, fractal dimension means space filling, spatial uniformity, and spatial complexity (Table 9).

### 4.3. Statistical Evaluation of Fractal Parameters

It is necessary to discuss fractal dimension measurement methods and related statistical test parameters simply. In practice, the double logarithmic linear regression based on the least square method can be employed to estimate fractal dimension values. Two methods can be utilized to carry out regression analysis: one is fixed intercept to 0, and the other is to let the intercept be free. The former can be termed fixed intercept regression, and the latter can be termed free intercept regression. For theoretical analysis, the intercept should be fixed to 0 so that the proportionality coefficient of the corresponding fractal model equals 1. For positive studies, the intercept depends on the measurement results and should not be fixed to a certain value [61]. No matter which method is adopted, a statistical test should be carried out for the calculation of results (Table 10). The basic and most important statistic for fractal dimension test is goodness of fit, *R*^2^, which is also termed the determination coefficient. Actually, the *R* statistic is called the multiple correlation coefficient, which equals the absolute value of the Pearson correlation coefficient for univariate linear regression analysis. Sometimes, we examine standard error and probability value, i.e., *p* value, of a fractal dimension. A proper statement in scientific research should be presented with a confidence statement [59]. A confidence statement comprises two elements: margin of error and level of confidence [62]. According to the standard error, *δ*, we can estimate the margin of error of a fractal dimension; according to the *p* value, we can calculate the level of confidence of the fractal dimension. In most cases, the fractal dimension calculation is based on univariate linear regression analysis. For univariate linear regression, the *R*^2^ value, the *F* statistic, *t* statistic, and the corresponding *p* value are equivalent to one another. What is more, the fractal dimension *D* and the *R*^2^ value can be associated with the standard error *δ*. The formulae are as follows (see Appendix A):

(14) $$F=t^{2}=\frac{vR^{2}}{1-R^{2}}$$

(15) $$\delta =D\sqrt{\frac{1/R^{2}-1}{v}}$$

where *v* denotes the degree of freedom. If the intercept of the log-log linear model for regression analysis is free, the degree of freedom is *v* = *n* − 2; if the intercept is fixed to 0, the degree of freedom is *v* = *n* − 1. Here, *n* is the sample size, i.e., the data point number. Then, using the *t* distribution function tdist, we can convert the *t* statistic into the corresponding *p* value by means of MS Excel. The grammar is “= tdist(abs(*t* value), *v*, 2)”. Thus based on the 95% level of confidence, the margin of error of the fractal dimension value can be approximately expressed as *D*±2*δ*.This means that, in the absence of special requirements, the *R*^2^ value will provide enough numerical information for statistical description of fractal dimension.

The analytical process and discussion of this paper are based on the standard definition of fractals. A fractal has three elements, i.e., form, chance, and dimension [63]. The first definition of Mandelbrot [4] (p. 15) based on dimension and chance is as follows: “A fractal is by definition a set for which the Hausdorff-Besicovitch dimension strictly exceeds the topological dimension.” The second definition based on form and chance is as follows: “A fractal is a shape made of parts similar to the whole in some way.” The second definition is given by Mandelbrot but published by Feder [3] (p. 11). The quantitative criterion of fractals is the Hausdorff–Besicovitch dimension. Recently, Jiang and his co-workers tried to relax the definition of fractals and gave the third definition as follows: a set or pattern is fractal if the scaling of far more small things than large ones recurs multiple times [64]. According to the new definition, the quantitative criterion of fractals is replaced by the head/tail index [65,66]: the ht-index of a fractal set or fractal pattern is at least three [64]. The new definition and criterion of fractals are very interesting and instructive. Sometimes, definitions of concepts or terms are most likely to lead to ambiguity, misunderstanding, and controversy. Therefore, scientific studies should sidestep the terminological minefield so that we can move beyond the semantic debate [67]. On the one hand, we should leave certain room for developing and consolidating a definition as the research approach continues to mature [67]. On the other hand, as West and West [68] (p. 210) once pointed out, “…science does not wait for definitions, it continues forward in exploring phenomena with or without a clear understanding, confident that such understanding will eventually emerge.” Saint Thomas Aquinas once said, “What, then, is time? If no one asks me, I know what it is. If I wish to explain it to him who asks me, I do not know.” Now, for me, what, then, is city/fractal/science? If no one asks me, I know what it is. If I wish to explain it to him who asks me, I do not know. Even so, as Potter Stewart, the well-known former judge of the United States, said, “I know it when I see it.” [12]. I know if it is a city when I see a city, I know if it is a fractal when I see a fractal, and I know if it is scientific research when I see a research result.

## 5. Conclusions

Fractal geometry provides us with a new mathematical framework of describing urban morphology. To characterize urban form and explain urban growth, we need various fractal dimensions. Fractal dimensions can be defined by generalized entropy and correlation functions. To understand the essence of fractal dimension, we must learn about entropy and correlation functions. On the one hand, fractal dimension is a characteristic value of entropy, and on the other, fractal dimension is a scaling exponent of correlation function. Where entropy is concerned, fractal dimension indicates uniformity, and inequality degree and uniformity degree represent two different sides of the same coin. In this sense, fractal dimension suggests difference and diversity. Where correlation is concerned, fractal dimension implies the complexity degree of dynamical systems. Moreover, to understand the concept of fractal dimension, we should know the notions of topological dimension and Euclidean dimension of embedding space in which fractal cities are defined. Fractal theory can be employed to carry out spatial analysis for the scale-free aspects of urban morphology. To research urban growth, we can employ sigmoid functions to model fractal dimension growth curves of urban form based on time series of fractal dimension. Thus, we have to know the upper limit and lower limit of fractal dimension values. The lower limit of fractal dimension relates to the topological dimension of fractal sets, while the upper limit depends on the embedding space dimension.

The main points of this paper can be summarized as follows. Firstly, fractal geometry is a powerful tool of scale-free analysis, and urban morphology is a typical scale-free geographical phenomenon. Therefore, fractal theory can be naturally applied to urban studies. Cities are not true fractals, but they can be treated as random pre-fractals, which bear fractal properties within certain scaling ranges. If urban form had characteristic scales, we would be able to calculate urban area and urban perimeters. Thus, urban form can be described with the methods from traditional advanced mathematics. Unfortunately, urban form has no characteristic scales: it belongs to scale-free distributions. A great many studies show that urban form follows power laws indicative of fractal nature. In this case, it is an advisable selection to employ fractal geometry to describe urban morphology and carry out scaling analysis of urban patterns and dynamic processes. Secondly, the most appropriate dimension of embedding space for city fractals is two dimensions rather than three dimensions. The upper limit of the fractal dimension of urban form should not exceed the embedding dimension. A city fractal can be defined in a two-dimensional space, and it can also be defined in a three-dimensional space. It is better to define city fractals in a two-dimensional space. On the one hand, fractal dimension is used to replace urban area, which cannot be objectively measured due to the scale-free distribution of cities. Urban area is a measure defined in a two-dimensional space. Therefore, city fractals can be defined in two-dimensional space so that fractal dimension can be employed to successfully replace urban area. On the other hand, the criterion of the scientific method is to reduce dimensions rather than increase dimensions. Moreover, more available datasets of cities are based on two-dimensional space. It is simpler and more effective to analyze a city fractal through two-dimensional space. Thirdly, the topological dimension of urban form is zero dimension rather than one dimension. The lower limit of fractal dimension is equal to or greater than the topological dimension. For modeling fractal dimension growth curves of urban form, it is significant to identify the lower limit of fractal dimension. In theory, urban form can be reduced to separated spaces, so the topological dimension of city fractals is *d*_T_ = 0. The lower limit of the fractal dimension of urban form is *D*_min_ = 0. The topological dimension of the urban boundary is 1, but the most important city fractals are based on the urban area instead of urban boundary. In practice, the lower limit of the fractal dimension of urban form can also be treated as *D*_min_ = 1, especially when the sample path is short. Based on a constant study area and fixed largest box, the lower limit of the fractal dimension of urban form should be taken as *D*_min_ = 0. Based on a variable study area and unfixed largest box, the lower limit of the fractal dimension of urban form should be taken as *D*_min_ = 1. Based on a constant study area, fixed largest box, and long sample path (time span is very large), the fractal dimension values of urban form are sometimes *D* < 1. The question of how to measure the *D*_min_ value depends on the concrete situation.

## Figures and Tables

**Table 1:** Three preconditions for understanding, developing, and generalizing fractal concepts.

Conditions	Formula	Note
Scaling law	$Tf(x)=f(\lambda x)=\lambda^{b}f(x)$	The relation between scale and the corresponding measures follow power laws.
Fractal dimension	$d_{T}<D<d_{E}$	The fractal dimension *D* is greater than the topological dimension *d*_T_ and less than the Euclidean dimension of the embedding space *d*_E_.
Entropy conservation	$\sum_{i=1}^{N(r)}P_{i}{}^{q}r_{i}^{(1-q)D_{q}}=1$	The Renyi entropy values of different fractal units (fractal subsets) are equal to one another.

**Note**: T—scaling transform; *x*—scale variable; *f*(*x*)—a function of *x*; *λ*—scale factor; *b*—scaling exponent; *D*—fractal dimension; *d*_T_—topological dimension; *d*_E_—Euclidean dimension of embedding space; *q*—order of moment; *P_i_*, *r_i_*—growth probability of the *i*th fractal set and its linear scale; *D_q_*—generalized correlation dimension; *N*(*r*)—number of fractal units with linear size *r*; *i*—ordinal number: *i* = 1,2,…, *N*(*r*).

**Table 2:** Two types of natural and social phenomena: scaleful and scale-free phenomena.

Type	Probability Distribution	Characteristics	Example	Mathematical Tools	Description
Scaleful phenomena (with characteristic scales)	Normal, exponential, logarithmic, lognormal, Weibull, etc.	We can find definite length, area, volume, density, eigenvalue, mean value, standard deviation, and so on.	Urban population density distribution, which follows exponential law	Traditional higher mathematics includes calculus, linear algebra, probability theory, and statistics.	Entropy function and Gaussian distribution
Scale-free phenomena (without characteristic scale)	Power law, various hidden scaling distributions	We cannot find effective length, area, volume, density, eigenvalue, mean value, standard deviation, and so on.	Urban traffic network density distribution, which follows power law	Fractal geometry, complex network theory, allometry theory, scaling theory	Fractal dimension and Pareto distribution

**Table 3:** Two approaches to defining the study area for fractal dimension estimation of urban form.

Approach	Property	Merit	Demerit	Dimension Range
Constant study area	Fixed size	The comparability of fractal parameters of different years is strong. The time series of fractal dimension can be used to reflect space replacement of urban region.	The reality of fractal parameters of each year is weak.	Between 0 and 2
Variable study area	Unfixed size	The reality of fractal dimension values of urban form is strong. The time series of fractal dimension can be used to reflect space filling of urban growth.	The comparability of fractal parameters of different years is weak.	Between 1 and 2

**Table 4:** A complete scientific research process consists of two elements.

Element	Level	Method	Purpose	Result	Finding	Fractal Theory
Description	Macro level	Mathematics, measurement, and computation	Data, numbers	Show characteristics of a system’s behavior	How a system works	Geometrical method
Understanding	Micro level	Observation, experience, experiments, and simulation	Insight, sharpen questions	Reveal dynamical mechanism	Why the system works in this way	Ideas of complex systems

**Table 5:** Two functions of fractal geometry in urban studies.

Function	Use	Purpose	Approach
Theoretical	Present postulates and produce models	Develop urban theory based on the possible world	Build mathematical models based on fractals or fractal dimension
Empirical	Process experimental and observational data	Solve practical problems in the real world	Rely heavily on fractal dimension

**Table 6:** Two types of models and methods of model building.

Model type	Property	Building Method	Principle	Example
Mechanistic model (structural model)	Theoretical model	Analytical method	Postulates and demonstration	Wilson’s spatial interaction model
Parametric model (functional model)	Empirical model	Experimental method	Data and fitting	Traditional gravity model

**Table 7:** Three approaches to developing models for fractal dimension growth curves of urban form.

Approach	Example and Mathematical Expression	Name
Produce new models	$D(t)=\frac{D_{\max}}{1+(D_{\max}/D_{\left(1\right)}-1)t^{-b}}$	Growth function of hidden scaling
	$D(t)=D_{\min}+\frac{D_{\max}-D_{\min}}{1+[(D_{\max}-D_{\left(1\right)})/(D_{\left(1\right)}-D_{\min})]t^{-b}}$	
Improve old model	$D(t)=\frac{D_{\max}}{1+(D_{\max}/D_{\left(0\right)}-1)e^{-kt^{2}}}$	Quadratic logistic function
Borrow model from another discipline	$D(t)=D_{\min}+\frac{D_{\max}-D_{\min}}{1+[(D_{\max}-D_{\left(0\right)})/(D_{\left(0\right)}-D_{\min})]e^{-kt}}$	Boltzmann equation

**Note**: (1) Models. The logistic function and Boltzmann equation of fractal dimension growth curve were demonstrated by Chen [34], and the quadratic logistic function was derived and demonstrated by Chen [35]. (2) Parameters. *D*(*t*)—fractal dimension of urban form at time *t*; *D*_(0)_—the initial value of fractal dimension of urban form (*t* = 0); *D*_max_, *D*_min_—the upper limit and lower limit of fractal dimension; *b*—the scaling exponent of fractal dimension growth; *r*—the original growth rate of fractal dimension.

**Table 8:** Four cases for the lower limit of fractal dimension growth curves of urban form.

	Fixed Study Area	Variable Study Area
In theory	*D*_min_ = 0, logistic function	*D*_min_ = 0, long sample path, logistic function; *D*_min_ = 1, usual cases, Boltzmann equation
In practice	*D*_min_ = 1, short sample path, Boltzmann equation; *D*_min_ = 0, usual cases, logistic function	*D*_min_ = 1, Boltzmann equation

**Table 9:** The three basic meanings of fractal dimension of urban morphology.

Basic Measurement	Principle	Meaning	Explanation
Degree of space filling	$\begin{array}{l} 2F=\frac{2\ln A_{b}(r)}{\ln A(r)}\\ =\frac{2\ln N_{b}(r)}{\ln N(r)}=D_{0} \end{array}$	Capacity dimension equals doubled space-filling ratio	The space-filling ratio equals the logarithm of occupied area divided by the logarithm of total area
Degree of spatial uniformity	$\begin{array}{l} 2F=\frac{2\ln H}{\ln H_{\max}}\\ =\frac{2\ln N_{b}(r)}{\ln N(r)}=D_{0} \end{array}$	Capacity dimension equals doubled normalized Hartley entropy	Entropy is a measure of spatial uniformity
Degree of spatial complexity	$C(r)=C_{1}r^{2(D_{0}-d)+1}$	Capacity dimension suggests a spatial correlation exponent	Spatial correlation indicates spatial complexity of cities

**Note**: The formula of space-filling degree is derived in this paper, and the spatial correlation function was presented by Chen [60]. Regarding the relationships between entropy and fractal dimension, see [59].

**Table 10:** The transformation relationships between *F* statistic, *t* statistic, *p* values, standard deviation, and fractal dimension.

Item	Free Intercept (Arbitrary Value)	Fixed Intercept (Zero)
**Fractal model**	$N(r)=Kr^{-D}$(0 < *K* < 2)	$N(r)=Kr^{-D}$(*K* = 1)
**Logarithmic linear relation**	lnN(r)=lnK−Dlnr	lnN(r)=−Dlnr (ln*K*=0)
**Degree of freedom**, ***v***	v=n−2	v=n−1
***F* statistic, *F*, *t* statistic, *t*, and goodness of fit, *R*^2^**	$F=t^{2}=\frac{(n-2)R^{2}}{1-R^{2}}$	$F=t^{2}=\frac{(n-1)R^{2}}{1-R^{2}}$
**Standard error *δ*, fractal dimension *D*, and *R*^2^**	$\delta =D\sqrt{\frac{1/R^{2}-1}{n-2}}$	$\delta =D\sqrt{\frac{1/R^{2}-1}{n-1}}$
**Margin of error of fractal dimension *D* (significance level *α*=0.05)**	D±2δ	D±2δ
**Excel conversion formula from *t* statistic to *p* value**	tdist(abs(t),n−2,2)	tdist(abs(t),n−1,2)
**Definition of *R* statistic**	Pearson correlation coefficient	Cosine coefficient

**Note**: (1) Fomulae. See Appendix A for derivation. (2) Parameters. *r*—spatial measurement scale such as linear size of box; *N*(*r*)—spatial measurement with linear size *r* such as the number of non-empty boxes; *K*—proportionality coefficient; *D*—fractal dimension; ln—natural logarithm function; *n*—sample size; *F*—*F* statistic; *t*—*t* statistic; *R*—multiple correlation coefficient; tdist, abs—MS Excel functions for *t* distribution and absolute value.

## Appendix A. Derivation of the Relationships between Fractal Dimension and Standard Error

The relationships between fractal dimension and standard error can be derived with the help of knowledge of linear algebra and statistics. The following regression coefficient formula and basic statistics such as correlation coefficient *R* and *F* and *t* statistics can be seen in many statistical analysis textbooks and will not be explained in detail. The fractal model can be expressed as a power function as below:

(A1) $$N(r)=Kr^{-D}$$

where *r* denotes the spatial measurement scale such as linear size of box, *N*(*r*) is the corresponding spatial measurement with linear size *r*, such as the number of non-empty boxes, *K* is the proportionality coefficient, and *D* is the fractal dimension. The natural logarithm of both sides of the Equation (A1) is

(A2) lnN(r)=lnK−Dlnr

Then, the relationships between fractal dimension and its standard error can be deduced in two cases.

(1) Free intercept regression. Suppose that *K* > 0 but *K* ≠ 1. Thus, we have free intercept regression. In this case, the degree of freedom is *v* = *n* − 2. For simplicity, Equation (A2) is expressed as a univariate linear regression equation as follows:

(A3) y=a+bx

in which *x* = ln(*r*) refers to the independent variable and *y* = ln*N*(*r*) to the dependent variable. As for the parameters, *a* = ln*K* denotes the intercept, and *b* = −*D* is the slope. The slope value is termed regression coefficient in linear regression analysis. By the idea of least error sum of squares, we can construct a normal equation system. Suppose the time of measurements is *n*, and the measurement sequence is numbered as *i* = 1, 2, …, *n*. Then, by using the Cramer rule, we can derive the formula of the regression coefficient:

(A4) $$b=\frac{\sum_{i=1}^{n}\left(x_{i}-\bar{x})(y_{i}-\bar{y}\right)}{\sum_{i=1}^{n}{\left(x_{i}-\bar{x}\right)}^{2}}$$

where $\bar{x}$,$\bar{y}$ represent arithmetic means of the independent variable *x* and dependent variable *y*, respectively. The multiple correlation coefficient is defined as

(A5) $$R^{2}=\frac{(\sum_{i=1}^{n}(x_{i}-\bar{x})(y_{i}-\bar{y}){)}^{2}}{\sum_{i=1}^{n}{\left(x_{i}-\bar{x}\right)}^{2}\sum_{i=1}^{n}{\left(y_{i}-\bar{y}\right)}^{2}}=\frac{\sum_{i=1}^{n}{\left({\hat{y}}_{i}-\bar{y}\right)}^{2}}{\sum_{i=1}^{n}{\left(y_{i}-\bar{y}\right)}^{2}}=1-\frac{\sum_{i=1}^{n}{\left(y_{i}-{\hat{y}}_{i}\right)}^{2}}{\sum_{i=1}^{n}{\left(y_{i}-\bar{y}\right)}^{2}}$$

Substituting Equation (A5) into Equation (A4) yields

(A6) $$b=\frac{\sum_{i=1}^{n}\left(x_{i}-\bar{x})(y_{i}-\bar{y}\right)}{\sqrt{\sum_{i=1}^{n}{\left(x_{i}-\bar{x}\right)}^{2}\sum_{i=1}^{n}{\left(y_{i}-\bar{y}\right)}^{2}}}\sqrt{\frac{\sum_{i=1}^{n}{\left(y_{i}-\bar{y}\right)}^{2}}{\sum_{i=1}^{n}{\left(x_{i}-\bar{x}\right)}^{2}}}=R\sqrt{\frac{\sum_{i=1}^{n}{\left(y_{i}-\bar{y}\right)}^{2}}{\sum_{i=1}^{n}{\left(x_{i}-\bar{x}\right)}^{2}}}$$

In fact, Equations (A4) and (A5) can be found in many textbooks of multiple statistical analyses. The *F* statistic is defined as

(A7) $$F=\frac{\sum_{i=1}^{n}{\left({\hat{y}}_{i}-\bar{y}\right)}^{2}}{\frac{1}{n-2}\sum_{i=1}^{n}{\left(y_{i}-{\hat{y}}_{i}\right)}^{2}}$$

Substituting Equation (A5) into Equation (A7) yields

(A8) $$F=\frac{(n-2)\sum_{i=1}^{n}{\left({\hat{y}}_{i}-\bar{y}\right)}^{2}/\sum_{i=1}^{n}{\left(y_{i}-\bar{y}\right)}^{2}}{\sum_{i=1}^{n}{\left(y_{i}-{\hat{y}}_{i}\right)}^{2}/\sum_{i=1}^{n}{\left(y_{i}-\bar{y}\right)}^{2}}=\frac{(n-2)R^{2}}{\left(1-R^{2}\right)}$$

The *t* statistic is defined as

(A9) $$t=\frac{b}{s}$$

where *s* refers to the standard error of the regression coefficient based on sample. In contrast, the standard error *δ* is based on population. The formula is

(A10) $$s=\sqrt{\frac{\frac{1}{n-2}\sum_{i=1}^{n}{\left(y_{i}-{\hat{y}}_{i}\right)}^{2}}{\sum_{i=1}^{n}{\left(x_{i}-\bar{x}\right)}^{2}}}$$

Equations (A7)–(A10) can be found in many statistics textbooks. Substituting Equations (A5), (A6), and (A10) into Equation (A9) yields

(A11) $$t=R\sqrt{\frac{\sum_{i=1}^{n}{\left(y_{i}-\bar{y}\right)}^{2}}{\sum_{i=1}^{n}{\left(x_{i}-\bar{x}\right)}^{2}}}/\sqrt{\frac{\frac{1}{n-2}\sum_{i=1}^{n}{\left(y_{i}-{\hat{y}}_{i}\right)}^{2}}{\sum_{i=1}^{n}{\left(x_{i}-\bar{x}\right)}^{2}}}=\sqrt{\frac{\sum_{i=1}^{n}{\left({\hat{y}}_{i}-\bar{y}\right)}^{2}}{\frac{1}{n-2}\sum_{i=1}^{n}{\left(y_{i}-{\hat{y}}_{i}\right)}^{2}}}=\sqrt{F}$$

Combining Equations (A8) and (A11), we have

(A12) $$F=t^{2}=\frac{(n-2)R^{2}}{\left(1-R^{2}\right)}$$

Substituting Equations (A9) into Equation (A12) yields

(A13) $$\frac{\left|b\right|}{s}=\sqrt{\frac{(n-2)R^{2}}{\left(1-R^{2}\right)}}$$

Considering the definition of slope given above, *b* = −*D*, we have

(A14) $$s=\left|b\right|\sqrt{\frac{1-R^{2}}{(n-2)R^{2}}}=D\sqrt{\frac{1/R^{2}-1}{n-2}}$$

Thus, based on the 95% level of confidence, corresponding to the significance level of 0.05, the margin of error of the fractal dimension value can be expressed as

(A15) $$D\pm \mathrm{tinv}(\alpha ,n-2)\cdot D\sqrt{\frac{1/R^{2}-1}{n-2}}\approx D(1\pm 2\sqrt{\frac{1/R^{2}-1}{n-2}})=D\pm 2s$$

where **tinv** is the MS Excel function for threshold value investigation of the *t* statistic, *α* denotes significance level. The grammar the function “tinv” is “tinv(*α*, *v*)”, and here the level of significance should be *α* = 0.05.

(2) Fixed intercept regression. If *K =* 1, then *a* = ln*K* = 0. Thus, we have fixed intercept regression. In this case, the degree of freedom is *v* = *n* − 1. Then, the Pearson coefficient is replaced by the cosine formula, that is

(A16) $$R^{2}=\frac{(\sum_{i=1}^{n}x_{i}y_{i}{)}^{2}}{\sum_{i=1}^{n}x_{i}^{2}\sum_{i=1}^{n}y_{i}^{2}}$$

Using the similar method, we can derive the relation between fractal dimension and standard error based on fixed intercept regression as below:

(A17) $$s=D\sqrt{\frac{1/R^{2}-1}{n-1}}$$

In order to save space, the detailed derivation of Equation (A17) is omitted. Readers can derive it by analogy with the process of deriving Equation (A14). Based on the 95% level of confidence, the margin of error of the fractal dimension value is

(A18) $$D\pm \mathrm{tinv}(\alpha ,n-1)\cdot D\sqrt{\frac{1/R^{2}-1}{n-1}}\approx D(1\pm 2\sqrt{\frac{1/R^{2}-1}{n-1}})=D\pm 2s$$

This means that, based on the 95% confidence level, the error margin of the fractal dimension is approximately the fractal dimension value plus or minus twice the standard error. Note that the sample standard error *s* here is used instead of the population standard error *δ* in the text. The population standard error *δ* is mainly for theoretical derivation, while the sample standard error *s* is principally for empirical analyses.
